# Supplementary material for: Use of an ETEC Proteome Microarray to Evaluate Cross-Reactivity of ETVAX® Vaccine-Induced IgG Antibodies in Zambian Children
Source: Vaccines (Basel). 2023 May 4;11(5):939. doi: 10.3390/vaccines11050939 (PMC10224314; doi:10.3390/vaccines11050939)
Supplement: Supplementary file 1 [file vaccines-11-00939-s001.zip › Etvax cross reactivity_MDPI_Supplementary table 1.pdf]

Table S1. General characteristics of study participants.

		Age (months)	Gender	ETEC results (colony PCR)		Sick Visit	
**ID**	**vaccine allocation**			**Visit 1**	**Visit 7**	**clinical diagnosis**	**severity**
S060-2002	1/8 dose	18	Female	Negative	Negative		
S064-2004	1/4 dose	13	Male	Negative	Negative		
S058-2007	1/8 dose	15	Male	Negative	Positive	Acute diarrhoeal disease with no dehydration	mild
S078-2010	Placebo	20	Male	Negative	Negative	acute gastroenteritis (with no dehydration)	moderate
S070-2013	Placebo	23	Female	Negative	Positive		
S085-2016	1/4 dose	18	Male	Negative	Positive		
S087-2019	Placebo	22	Female	Positive	Negative		
S094-2022	1/4 dose	14	Male	Negative	Negative		
S095-2025	1/8 dose	19	Female	Negative	Negative		
S104-2028	1/4 dose	21	Female	Negative	Positive		
S103-2031	1/8 dose	14	Female	Negative	Negative	Acute Gastroenteritis	moderate
S114-2034	1/4 dose	23	Female	Negative	Negative		
S107-2037	1/8 dose	21	Female	Negative	Positive	acute gastroenteritis with no dehydration	moderate
S125-2040	1/4 dose	17	Female	Negative	Positive		
S129-2043	Placebo	23	Female	Negative	Negative		
S131-2046	1/4 dose	18	Female	Negative	Negative	Acute gastroenteritis with severe dehydration	severe
S147-2049	1/8 dose	21	Female	Negative	Negative		
S145-2052	1/4 dose	20	Female	Positive	Negative		
S148-2055	1/8 dose	17	Female	Negative	Negative	acute gastroenteritis with no dehydration	moderate
S142-2057	1/8 dose	16	Male	Positive	Positive	acute gastroenteritis with no dehydration	moderate
