# Supplementary material for: Use of an ETEC Proteome Microarray to Evaluate Cross-Reactivity of ETVAX® Vaccine-Induced IgG Antibodies in Zambian Children
Source: Vaccines (Basel). 2023 May 4;11(5):939. doi: 10.3390/vaccines11050939 (PMC10224314; doi:10.3390/vaccines11050939)
Supplement: Supplementary file 1 [file vaccines-11-00939-s001.zip › Etvax cross reactivity_MDPI_Supplementary table 2.pdf]

Table S2: Description of purified proteins included in the microarray.

Array ID	Full-length protein name	Description
C-EtpA 0.1	EtpA	C-EtpA
CFA/I 0.1	CFA/I	CFA/I: Native fimbriae [WS1933D]
CFA/I lot BB-143 0.1	CFA/I	CFA/I: (lot BB 143) Native fimbriae [WS1933D]
CfaEad[His]6 0.1	CFA/I	p CFA/I: CfaE adhesin domain truncate [E7473/0]3
CotDad[His]6 0.1	CS2	p CS2: CotD adhesin domain truncate [C91f]
CS1 0.1	CS1	CS1: native fimbriae [3775a-1(pRA101)]
CS14 0.1	CS14	CS14: native fimbriae [WS3294A(pRA101)]
CS17 0.1	CS17	CS17: native fimbriae [LSN03-016011/A]
CS19 0.1	CS19	CS19: native fimbriae [WS0115A(pRA101)]
CS2 0.1	CS2	CS2: native fimbriae [C91f]
CS3 0.1	CS3	CS3: native fimbriae [E9034A]
CS3 lot BB-107B 0.1	CS3	CS3: native fimbriae [E9034A]
CS4 0.1	CS4	CS4: native fimbriae [WS2589A]4
CS6 0.1	CS6	CS6: recomb. fimbriae [E8775]
CS6 lot BB-063B 0.1	CS6	CS6: recomb. fimbriae [E8775]
CsbDad[His]6 0.1	CS17	p CS17: CsbD adhesin domain truncate [WS6788A]
dsc15CotD[His]6(GGG) 0.1	CS2	p CS2: minor subunit (adhesin) [C91f]
dsc15CsfD[His]6 0.1	CS4	p CS4: minor subunit (adhesin) [WS2560B]
dsc16ACssA 0.1	CS6	p CS6: major subunit [E8775]
dsc16BCssB 0.1	CS6	p CS6: major subunit [E8775]
dsc16GCstG[His]6 0.1	CS3	p CS3: major subunit (pilin) [M424C1]
dsc16GCstH[His]6 0.1	CS3	p CS3: major subunit (pilin) [M424C1]
dsc19CfaB[His]6 0.1	CFA/I	p CFA/I: major subunit (pilin) [E7473/0]
dsc19CfaE[His]6 0.1	CFA/I	p CFA/I: minor subunit (adhesin) [E7473/0]
dsc19CsbD[His]6(GGG) 0.1	CS17	p CS17: minor subunit (adhesin) [LSN02-013966/A]
dsc19CsuD[His]6 0.1	CS14	p CS14: minor subunit (adhesin) [WS3294A]
dscCooA 0.1	CS1	p CS1: major subunit (pilin) [E24377A]
dscCooD[His]6 0.1	CS1	p CS1: minor subunit (adhesin) [E24377A]
dsCotA 0.1	CS2	p CS2: major subunit (pilin) [C91f]
EaeH 0.1	EaeH	EaeH
EatA 0.1	EatA	EatA
EtpA 0.0001	EtpA	EtpA 0.0001
FimHLD 0.1	FimHLD	FimHLD
LT-A 0.1	LT-A	LT-A
LT-B 0.1	LT-B	LT-B
N-EtpA 0.1	EtpA	p N-EtpA
PCFO71 0.1	PCFO71	PCFO71: native fimbriae [WS2173A]
YghJ 0.1	YghJ	YghJ
