# Supplementary material for: Use of an ETEC Proteome Microarray to Evaluate Cross-Reactivity of ETVAX® Vaccine-Induced IgG Antibodies in Zambian Children
Source: Vaccines (Basel). 2023 May 4;11(5):939. doi: 10.3390/vaccines11050939 (PMC10224314; doi:10.3390/vaccines11050939)
Supplement: Supplementary file 1 [file vaccines-11-00939-s001.zip › Etvax cross reactivity_MDPI_Supplementary table 3.pdf]

Table S3: Group averages for the top 10 antigens ranked by absolute mean difference in the all-vaccinated group.

Antigen	Species	Description	V1 Average (N=16)	V7 Average (N=16)	Average Difference	T-Test P-Value	AUC	Wilcox P-Value	Increase 50 Pct Count	Increase 50 Pct Frequency
CFA/I lot BB–143 0.1	ETEC	CFA/I lot BB–143 0.1	11.65	13.79	2.15	1.1e–02	0.68	9.4e–02	10	0.62
CS6 lot BB–063B 0.1	ETEC	CS6 lot BB–063B 0.1	10.10	12.19	2.08	1.4e–02	0.70	5.6e–02	7	0.44
CS4 0.1	ETEC	CS4 0.1	9.27	11.08	1.82	4.3e–02	0.66	1.4e–01	10	0.62
CFA/I 0.1	ETEC	CFA/I 0.1	9.57	11.37	1.81	6.1e–03	0.75	1.4e–02	10	0.62
CS6 0.1	ETEC	CS6 0.1	9.94	11.52	1.58	6.2e–02	0.62	2.4e–01	6	0.38
dsc16GCstG[His]6 0.1	ETEC	dsc16GCstG[His]6 0.1	8.34	9.78	1.44	1.2e–02	0.71	4.8e–02	10	0.62
LT-B 0.1	ETEC	LT-B 0.1	11.17	12.49	1.32	4.4e–05	0.83	1.1e–03	14	0.88
dsc16GCstH[His]6 0.1	ETEC	dsc16GCstH[His]6 0.1	10.33	11.64	1.31	6.5e–03	0.69	6.1e–02	8	0.50
PCFO71 0.1	ETEC	PCFO71 0.1	9.41	10.71	1.30	3.9e–04	0.77	8.8e–03	12	0.75
CS14 0.1	ETEC	CS14 0.1	9.73	11.00	1.27	2.8e–02	0.68	9.4e–02	6	0.38
