# Supplementary material for: Use of an ETEC Proteome Microarray to Evaluate Cross-Reactivity of ETVAX® Vaccine-Induced IgG Antibodies in Zambian Children
Source: Vaccines (Basel). 2023 May 4;11(5):939. doi: 10.3390/vaccines11050939 (PMC10224314; doi:10.3390/vaccines11050939)
Supplement: Supplementary file 1 [file vaccines-11-00939-s001.zip › Etvax cross reactivity_MDPI_Supplementary table 4.pdf]

Table S4: Delta changes in group averages for the top 10 antigens in all vaccinated individuals compared to the placebo group at visit 1 and visit 7.

Antigen	Species	Description	Placebo Average (N=4)	All.Vaccinated Average (N=16)	Average Difference	T-Test P-Value	AUC	Wilcox P-Value
CS6 lot BB–063B 0.1	ETEC	CS6 lot BB–063B 0.1	–0.362	2.085	2.45	1.9e–02	0.73	1.8e–01
CFA/I lot BB–143 0.1	ETEC	CFA/I lot BB–143 0.1	–0.099	2.147	2.25	9.8e–03	0.83	5.0e–02
CFA/I 0.1	ETEC	CFA/I 0.1	–0.033	1.806	1.84	7.8e–03	0.81	6.4e–02
CS6 0.1	ETEC	CS6 0.1	–0.252	1.579	1.83	3.5e–02	0.67	3.4e–01
dsc16GCstH[His]6 0.1	ETEC	dsc16GCstH[His]6 0.1	–0.080	1.308	1.39	9.7e–02	0.80	8.0e–02
CfaEad[His]6 0.1	ETEC	CfaEad[His]6 0.1	–1.119	0.189	1.31	6.3e–02	0.86	2.9e–02
dsc16BCssB 0.1	ETEC	dsc16BCssB 0.1	0.001	1.259	1.26	7.7e–02	0.64	4.4e–01
CS19 0.1	ETEC	CS19 0.1	1.323	0.073	–1.25	2.1e–01	0.25	1.5e–01
dsc16GCstG[His]6 0.1	ETEC	dsc16GCstG[His]6 0.1	0.204	1.443	1.24	3.6e–02	0.70	2.5e–01
CS17 0.1	ETEC	CS17 0.1	1.048	–0.042	–1.09	3.6e–01	0.33	3.4e–01
