# Supplementary material for: Use of an ETEC Proteome Microarray to Evaluate Cross-Reactivity of ETVAX® Vaccine-Induced IgG Antibodies in Zambian Children
Source: Vaccines (Basel). 2023 May 4;11(5):939. doi: 10.3390/vaccines11050939 (PMC10224314; doi:10.3390/vaccines11050939)
Supplement: Supplementary file 1 [file vaccines-11-00939-s001.zip › Etvax cross reactivity_MDPI_Supplementary table 5.pdf]

Table S5: Plasma antibody responses after 2, 3, and 2 or 3 doses (≥4-fold and ≥2-fold and ≥2 or 4-fold) in cohort B children 10–23 months given 1/8 and 1/4 dose of ETVAX + dmLT or placebo (OEV124).

Group	C2FA/I			CS3			CS5			CS6			LTB IgA			LTB IgG		
	2	3	2or3	2	3	2or3	2	3	2or3	2	3	2or3	2	3	2 or3	2	3	2or3
V 1/8 dose n=20<br>≥ 4-fold resp.	1	4	5	4	6	8	1	4	5	1	2	3	12	13	15	15	13	
≥ 2 fold resp.	6	7	9	5	3	5	3	0	3	3	3	5	6	2	4	3	5	
V1/4 dose n=19<br>≥ 4-fold resp.	4	8	9	2	4	4	3	6	6	0	0	0	8	10	11	6	8	
≥ 2-fold resp.	2	1	2	4	2	5	2	1	2	1	2	2	5	3	3	6	5	
Total no of V responders (%)	13	20	25	15	15	22	10	10	16	5	6		31	28	33	31	29	
	25/39 **(64%)**			22/39 **(56%)**			16/39**(41%)**			10/39 **(26%)**			33/39 **(85%)**			32/37**(86%)**		
Placebos n=19<br>Pl ≥ 4-fold resp.	0	2		1	1		2	3		2	3		4	8		4	8	
Pl ≥ 2-fold resp.	6	6		4	2		0	3		1	2		1	4		1	3	
Total no of P responders (%)	13/20 **(65%)**			7/20 **(35%)**			8/20 **(42%)**			6/20 **(30%)**			12/20 **(60%)**			12/19 **(63%)**		
**V vs P all responders**	**ns**			**ns**			**ns**			**ns**			**p=0.052**			**p=0.08**		
